# Intelligent Treatment of Domestic Animals

**Published:** 1893-08

**Authors:** 


### EDITORIAL DEPARTMENT.

F. E. DANIEL, M. D., Editor.
S. E. HUDSON, M. D., Managing Editor.
A. J. SMITH. M. D., Galveston, Associate Editor.

EDITORIAL STAFF:

PROF. J. E. THOMPSON, M. D., Texas Medical College, Galveston; Surgery.
PROF. WM. KEILLER, M. D., Texas Medical College, Galveston; Obstetrics and Gynecology.
PROF. DAVID CERNA, M. D., Texas Medical College, Galveston; Therapeutics.
PROF. A. J. SMITH, M. D., Texas Medical College, Galveston; Medicine.
DR. ISADORE DYER, Tulane University; Dermatology.
DR. R. H. L. BIBB, Saltillo, Mexico; Foreign Correspondent.
DR. ROBT. MORRIS, Charity Hospital, N. O.; Clinical Reports.

——o——

The JOURNAL is the official organ of the Austin District Medical Society, the West Texas District Medical Society and the Galveston County Medical Society.

### INTELLIGENT TREATMENT OF DOMESTIC ANIMALS

Readers of the JOURNAL will remember that we have ever advocated a more intelligent and rational treatment of our domestic animals than that which, except in certain favored localities, they get at the hands of so-called veterinary surgeons. In Europe, and especially in Germany, it requires a long course of careful study to enable one to become a horse doctor,—the anatomy and physiology of the quadrupeds are as carefully studied as that of the biped animal in this country, and clinics where pathology and treatment are taught, are as common as the other kind of clinics are here. But in America, anybody, especially one who has kept a livery stable, or clerked in one, can become a "veterinary surgeon." Speaking in general terms, their knowledge of anatomy, physiology and pathology, as well as of materia medica and therapeutics, is *nil*, while their entire knowledge of treatment consists of a few drenches or washes, handed down from one generation to another, and entirely empirical; and their surgical resources consist of bleeding from the roof of the mouth, or blowing up the skin for "sweeney," or burning out the swollen gum with a hot iron for that condition known as the "lampers." Of course there are many exceptions, but they are confined to cities,—and a ten thousand dollar race horse or a thorough-bred cow, bull or sheep has a slim chance for his life should he get really sick, or meet with a barbed-wire-fence or other accident; the only resource is the horse doctor at the nearest livery stable, with his drenches, etc.

The JOURNAL makes a plea in behalf of the valuable, patient dumb servants; we think that humanity demands that physicians give some attention to diseases of the horse, dog and cow, and when called on give them the benefit of their skill and knowledge, the same as to other patients. Why not? It is a noble study, and, leaving out all humanitarian considerations, it can be made remunerative. The owner of a three hundred dollar mule would gladly pay ten or twenty dollars to a surgeon to sew up a cut, arrest a hemorrhage, or render similar service whereby the life and usefulness of the animal may be preserved. Why not prescribe for, or perform an operation on a horse or a dog or a cow, or even a hog, if requested to do so, and why not render a bill for and take pay for the service? We see no reason why not, but many reasons why yes.

The foregoing reflections have been brought out by seeing the following card, sent us by Dr. Talley, of Temple. Dr. Talley is President of the Austin District Medical Society, and an old member of the State Medical Association. He is a great lover of fine stock,—and being a humane man, as well as an accomplished physician and surgeon, and recognizing a real want in his section—a fine stock-raising country—for skilled knowledge and service for stock, he has very sensibly undertaken to practice veterinary medicine and surgery. He has taken the initiative step in a very sensible and important movement, one which we hope to see numerously followed by physicians all over the State.

The JOURNAL gives the doctor's card free insertion, and commends his good sense, wishing him success in his new field of laudable labor:

VETERINARY AND BOARDING STABLE,

Avenue B, Between 12th and 14th Sts., for the

CARE AND TREATMENT OF ALL DOMESTIC ANIMALS.

*R. P. Talley, M. D., Proprietor.*

TELEPHONE.

TEMPLE, - - - TEXAS.

To accommodate some friends, I can be found at the Central Hotel, at Belton, Texas, every Wednesday.

[Reverse side of card.]

"It has been, and no doubt will be, said by the uninformed, that I have abandoned the practice of medicine and surgery for the human family. Such is not intended by the publication of this card, but when not so engaged I will serve my friends in the interest of their domestic animals as best I can, for which I shall charge fees as may seem right to me, but never more, and generally less, than for like services for the human family. In the practice of medicine and surgery for our domestic animals, I am rendering service to our uncomplaining servants, as well as thereby becoming, from day to day, better able to render good service to the human animal, the domestic and other inferior animals having furnished uncomplainingly perhaps three-fourths of our present reliable information regarding the ills to which human flesh is heir.

"The faithful family physician cannot be found who is not willing to advise his patrons for the welfare of their pet stock and domestic animals, and why should they not pay for such service as well as for anything else of value? My stable is especially arranged for the comfort and health of all animals in my charge.

"For pleasure and profit, I am breeding two species of the dog family—English Mastiff and Bloodhounds, of the best blood to be found in any country."

